# Prognostic survival model for people diagnosed with invasive cutaneous melanoma

**DOI:** 10.1186/s12885-015-1024-4

**Published:** 2015-01-31

**Authors:** Peter D Baade, Patrick Royston, Philipa H Youl, Martin A Weinstock, Alan Geller, Joanne F Aitken

**Affiliations:** 1 Cancer Council Queensland, 553 Gregory Terrace, Fortitude Valley, Queensland Australia; 2 School of Public Health and Social Work, Queensland University of Technology, Brisbane, Queensland Australia; 3 Griffith Health Institute, Griffith University, Gold Coast, Queensland Australia; 4 MRC Clinical Trials Unit at University College London, London, UK; 5 Dermatoepidemiology Unit, V A Medical Center, Providence, RI USA; 6 Department of Dermatology, Rhode Island Hospital, Providence, RI USA; 7 Departments of Dermatology and Epidemiology, Brown University, Providence, RI USA; 8 Harvard School of Public Health, Harvard University, Boston, MA USA

**Keywords:** Melanoma, Survival, Prognostic model, Thickness, Population-based, Risk

## Abstract

**Background:**

The ability of medical practitioners to communicate risk estimates effectively to patients diagnosed with melanoma relies on accurate information about prognostic factors and their impact on survival. This study reports the development of one of the few melanoma prognostic models, called the Melanoma Severity Index (MSI), based on population-based cancer registry data.

**Methods:**

Data from the Queensland Cancer Registry for people (20–89 years) diagnosed with a single invasive melanoma between 1995 and 2008 (n = 28,654; 1,700 melanoma deaths). Additional clinical information about metastasis, ulceration and positive lymph nodes was manually extracted from pathology forms. Flexible parametric survival models were combined with multivariable fractional polynomial for selecting variables and transformations of continuous variables. Multiple imputation was used for missing covariate values.

**Results:**

The MSI contained the variables thickness (transformed, explained 40.6% of variation in survival), body site (additional 1.9% in variation), metastasis (1.8%), positive nodes (0.7%), ulceration (1.3%), age (1.1%). Royston and Sauerbrei’s D statistic (measure of discrimination) was 1.50 (95% CI = 1.44, 1.56) and the corresponding RD2 (measure of explained variation) was 0.47 (0.45, 0.49), demonstrating strong explanatory performance. The Harrell-C statistic was 0.88 (0.88, 0.89). Lacking an external validation dataset, we applied internal-external cross validation to demonstrate the consistency of the prognostic information across geographically-defined subsets of the cohort.

**Conclusions:**

The MSI provides good ability to predict survival for melanoma patients. Beyond the immediate clinical use, the MSI may have important public health and research applications for evaluations of public health interventions aimed at reducing deaths from melanoma.

## Background

The incidence of cutaneous melanoma in Australia is the highest in the world due to the combination of high ultraviolet radiation, outdoor lifestyle and predominately Caucasian population [1]. As most melanomas in Australia are diagnosed when thin, [2,3] overall survival from melanoma is high. Internationally, overall five-year survival estimates in Western countries exceed 85% [3-6], although they are lower in Eastern Europe [7]. However there remains an important subset of melanomas that are diagnosed at an advanced stage, many with clinically apparent metastatic spread, for which the prognosis is poor [3,8,9].

Prognostic models are used to assist clinicians with their assessment of a patient’s future outcome and so enhance their informed decision making process with the patient [10]. Most prognostic models for melanoma are derived from selected clinical cohorts composed of patients referred to tertiary hospitals or specialist cancer centres [11,12] and/or focussed on specific subgroups of melanomas such as localised [13] or nodular melanoma [14]. A similar limitation in regards to specific cohorts of melanoma patients holds for population-based studies of survival outcomes [2,15,16].

We describe here the development of a prognostic model for deaths from melanoma among all patients diagnosed with a single invasive cutaneous melanoma in Queensland, Australia, using data from a population-based cancer registry, and discuss the potential application in clinical practice and research.

## Methods

Ethics approval to conduct this study was obtained from the University of Queensland Behavioural & Social Sciences Ethical Review Committee. Since these data are not publically available, approval to access the required data was obtained from the Queensland Department of Health.

Data were obtained from the Queensland Cancer Registry (QCR) for all patients in Queensland with a histologically confirmed diagnosis of invasive melanoma (C44, M872-879) for the period 1995 to 2008. Notification of all cancers, apart from squamous and basal cell cancer, is required by law. Melanomas diagnosed on the basis of metastasis only were excluded. Variables extracted for each patient were sex, age at diagnosis, date of diagnosis, anatomic sub-site of the melanoma, tumour thickness, (Clarks) level of invasion and tumour morphology. Information on ulceration, presence and extent of metastasis at diagnosis and the number of positive lymph nodes (local, regional or distal)at diagnosis was extracted from pathology forms held by the Registry.

Due to the difficulty of attributing death to a specific melanoma, we excluded all patients who were known to have had more than one histologically confirmed diagnosis of invasive melanoma since the establishment of the QCR in 1982. As with a previous report [2] only patients aged 15–89 years at diagnosis were initially considered as there is evidence that melanoma survival outcomes are different for younger age groups [17] and death certificates are less precise for older patients [18].

The QCR database was matched against the Queensland Register of Births, Deaths and Marriages and the National Death Index to identify deaths in Queensland and interstate respectively up to 31st December 2010. Cause of death was coded using all available information from death certificates, autopsy reports and pathology reports.

Melanoma-specific survival was estimated from the mortality of people diagnosed with melanoma between 1995 and 2008 (inclusive), with follow-up for all cases to December 31, 2010. Patients who died before 31^st^ December 2010 from conditions other than melanoma were censored at date of death. Those who were not recorded as dying by 31^st^ December 2010 were censored at this date.

Median follow-up time was calculated using the reverse Kaplan-Meier method [19]. All data analyses were performed using Stata/SE version 12.1 for Windows (StataCorp, TX, USA). The Royston-Parmar models were fitted using the stpm2 package [20,21].

### Multiple imputation by chained equations

Due to missing values in the included variables (Table 1), a complete case analysis would have excluded at least 30% of the initial cohort, potentially introducing a bias if the excluded cases were a non-random sample.

**Table 1:** **Cause-specific survival: invasive Melanoma in Queensland (1995–2008, follow-up to 2010)**

	Total	1 year survival	5 year survival	10 year survival
Total	28,654 (100%)	99.1	95.0	92.6
Gender				
Male	16,269 (57%)	98.9	93.8	90.8
Female	12,385 (43%)	99.4	96.5	94.8
Age group (years)^a^				
20-39	4,958 (17%)	99.8	97.4	96.1
40-59	10,814 (38%)	99.5	96.5	94.4
60-69	5,405 (19%)	99.1	94.6	91.7
70-79	5,006 (17%)	98.7	92.0	87.9
80-89	2,471 (9%)	97.5	88.6	85.2
Thickness^a^				
<=0.50 mm	12,641 (44%)	100	99.6	99.0
0.51-1.00	8,107 (28%)	99.9	97.9	96.2
1.01-1.50	2,487 (9%)	99.2	93.1	88.3
1.51-2.00	1,386 (5%)	98.8	87.9	80.3
2.01-4.00	2,234 (8%)	97.3	79.6	70.8
4.01+	1,170 (4%)	92.9	67.8	61.4
Unknown^b^	629 (2%)	91.5	82.6	78.6
Body site				
Scalp	565 (2%)	96.2	78.8	69.3
Face/Lip/Eyelid	2,221 (8%)	99.0	94.5	90.4
Ear/Neck	1,741 (6%)	98.9	93.4	90.6
Chest/Axilla	1,435 (5%)	99.2	96.0	94.0
Abdomen + Hip	791 (3%)	98.7	93.7	90.7
Back/Buttocks	8,046 (28%)	99.5	95.2	92.8
Upper arm/Forearm/Hand/Finger/sb.hand	3,644 (13%)	99.5	96.6	95.4
Foot/sb. foot/heel/toe^c^	384 (1%)	97.4	87.7	79.6
Thigh/leg/ankle	5,961 (21%)	99.5	96.5	94.7
Shoulder	2,452 (9%)	99.5	96.0	93.7
Unknown^b^	1,414 (5%)	96.4	92.0	90.2
Morphology				
Superficial spreading melanoma	16,229 (57%)	99.7	97.3	95.7
Nodular melanoma	2,415 (8%)	96.2	79.0	73.1
Malignant melanoma in junctional naevus	654 (2%)	99.7	98.0	97.1
Lentigo Maligna melanoma	1,688 (6%)	99.8	97.4	95.6
Acral lentiginous melanoma	144 (<1%)	97.9	85.5	80.2
Other specified melanoma	1,015 (4%)	97.5	87.6	79.8
Malignant melanoma NOS	6,509 (23%)	98.9	95.2	92.7
Ulceration				
No ulceration	17,110 (60%)	99.7	97.3	95.5
Known ulceration	2,627 (9%)	95.4	77.0	70.7
No mention on pathology^b^	8,917 (31%)	99.1	95.5	93.1
Clarks level				
Into papillary dermis	15,488 (54%)	99.9	99.4	98.5
Filling papillary dermis	6,164 (22%)	99.6	95.3	92.7
Into reticular dermis	5,584 (19%)	98.0	86.6	80.3
Into subcutaneous fat	715 (3%)	93.4	70.0	60.7
Unknown^b^	703 (2%)	92.4	80.9	73.9
Positive nodes				
None	28,421 (99%)	99.3	95.4	93.0
At least one	229 (<1%)	75.2	43.6	37.5
Metastasis				
No	28,506 (99%)	99.3	95.3	92.9
Yes	148 (<1%)	68.7	39.4	27.6

Note: ^a^Modeled as a continuous variable in the prognostic model.

^b^Actual values for missing data were estimated in the modeling process using multiple chained imputation.

^c^sb = subungal; NOS = Not otherwise specified.

We used multiple imputation [22] methods to deal with the missing data, using the *mi impute chained* and *mi estimate* commands for chained equations and subsequent regression model estimation. In the imputation modelling we included the Kaplan-Meier estimate of the survival curve, vital status, and the interaction between the survival and vital status. All variables included in the prognostic model were also included in the series of chained imputation equations. We used n = 30 imputations based on the percentage of incomplete cases [23]. Predictive mean matching was used for the imputation of thickness, logit models for lymph nodes (none vs one or more) and ulceration (yes, no), and multinomial logit models for Clark’s level and subsite.

### Derivation of the survival model

Kaplan-Meier survival estimates, stratified by each of the covariates, were calculated to describe the melanoma cohort. We then developed a multivariable survival model to generate the prognostic index. Multivariable survival analyses are generally carried out using Cox regression. However several authors have highlighted the limitations of this method for prognostic models, [21,24] particularly relating to the appropriate modelling of the baseline hazards function.

A parametric alternative to the Cox model, known as a flexible parametric survival model, is fitted on the log cumulative hazard scale [20,21]. These Royston-Parmar (RP) models use natural cubic splines to estimate the baseline cumulative hazard function. The selection of scales and number of degrees of freedom for the baseline spline function was made based on the Bayes information criterion (BIC) statistic. For our data, the probit scale with 3 degrees of freedom provided the best fit. These 3 degrees of freedom equate to 2 interior knots along with the 2 boundary knots.

We then ran a multivariable fractional polynomial (MFP) procedure designed for multiple imputed data to help guide the backwards selection of covariates, and identify appropriate transformation(s), to retain in the fully adjusted survival model [25,26]. To improve the fit, we incorporated an additional smooth rank transformation [27] which accommodates sigmoid dose–response relationships. Visual inspection of the smoothed martingale residuals for the regression models was also used to assess and revise the adequacy of the final transformations of continuous covariates.

Finally, we used a forward selection process to investigate whether the regression coefficients for any variables depended on follow-up time using the approach described by Royston & Parmar [20,21] with one degree of freedom for the time dependent covariate effects. This approach modifies the spline function of time used to model the baseline distribution function and is implemented using the stpm2t command in Stata. Covariates without time dependence were included in the model in standard fashion.

### Discrimination

The discrimination of a prognostic model reflects its ability to distinguish between patient outcomes, and is closely related to the idea of the proportion of variance that the model explains. We calculated Royston and Sauerbrei’s D statistic [28] as a measure of discrimination, and ${\mathrm{R}}_{\mathrm{D}}^2$ as a measure of explained variation on the natural scale of the model [28]. We also calculated the Harrel’s C discrimination index, which is a scored on a scale of 0 to 1. This can be taken to mean that if two cases are drawn at random, the c statistic is the probability that the person who survives the longest had the highest predicted survival. Values near 0.5 suggest the prognostic score is equivalent to a coin toss in determining which patient will live longer, while values near 0 or 1 indicate perfect discrimination.

We assessed the importance of each variable in the prognostic model by examining the impact that removing the variable from the model had on D and ${\mathrm{R}}_{\mathrm{D}}^2$, both by removing just one of the variables, and then by sequentially removing the variables from the model [28].

### Development of the melanoma severity index (MSI)

While a complete prognostic model containing all significant covariates may have its advantages, in particular being able to explain the greatest amount of variation in survival, a parsimonious model containing only important predictors may be preferred for clinical application [29]. To develop this reduced model, known as the MSI, we based the variable selection on the BIC statistics and the reduction in the per cent of explained variation when the variable was removed from the model [29,30].

For illustration, predicted survival probabilities and survival curves, along with their 95% confidence intervals were then calculated from the MSI based on specific combinations of the final set of prognostic variables.

### Validation and calibration

Calibration reflects prediction accuracy. A well-calibrated prognostic model assigns the correct mean survival probability at all levels of predicted risk. We used an internal-external cross validation (IECV) method [31] for validating and assessing the calibration of the MSI.

**Figure 1:**
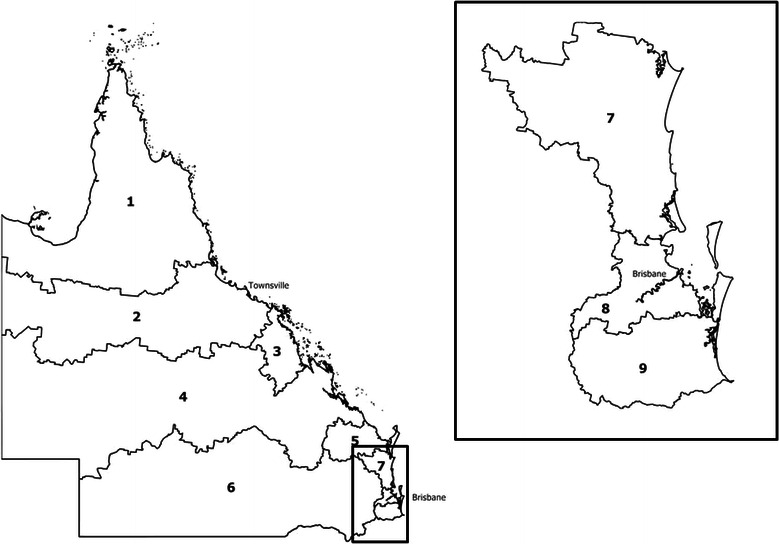
**Regions for internal-external validation, Queensland, Australia.**

Briefly, the IECV method involves splitting Queensland into 9 geographical regions (Figure 1). The MSI is then fitted using data from eight of these regions. The linear predictor, Xβ was estimated and applied to both the fitted data (eight regions, “k”) and the excluded data (remaining 9^th^ region, “(k)”). Values of Royston and Sauerbrei’s D statistic [28] were calculated from each result (D_k_ and D_(k)_ respectively). If the predictive ability of the model is maintained, both values of D will be approximately equal. The difference between the two values (d_k_) was calculated, with its standard error being the square root of the sum of the squared standard errors for D_k_ and D_(k)_. This process was repeated across the nine geographical regions.

Using these results, we assessed the calibration by comparing the predicted mean survival curves in each geographical region with the observed Kaplan-Meier survival estimates in that region.

## Results

### Patient characteristics

Of 35,264 patients diagnosed with invasive melanoma in Queensland between 1995 and 2008, 34,384 were aged between 20 to 89 years at diagnosis, and 28,654 had no other histologically confirmed invasive melanoma diagnosed since 1982 (Table 1). The median follow up time, calculated as the median time to censoring, was 7.2 years. A total of 5,469 (19%) had died before the 31^st^ December 2010. Almost a third of these deaths (31%) were ascribed to melanoma.

### Multivariable analysis

**Figure 2:**
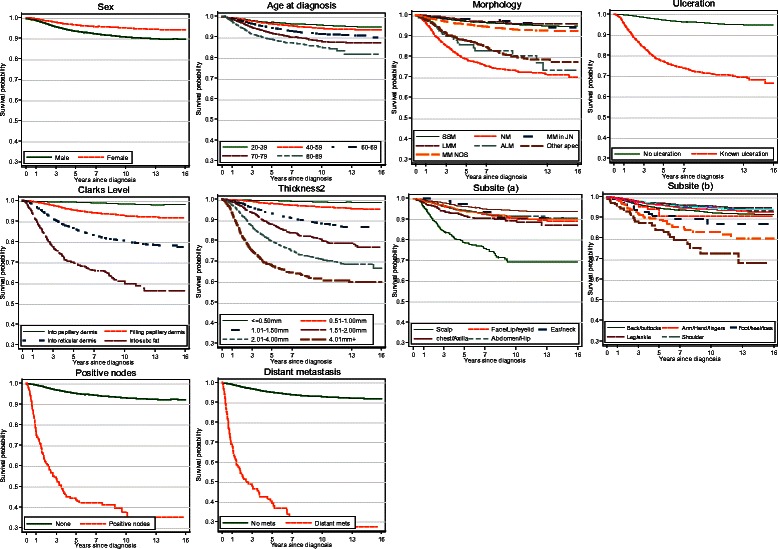
**Kaplan-Meier survival estimates by covariate group (1995–2008, follow up to 2010).**

All variables shown in Table 1 were considered in the initial prognostic model (see Figure 2). There was some evidence that sub-site had a time-dependent regression coefficient, so that the survival differential by subsite varied on the probit scale by follow up interval. However the effect was small and of no clinical relevance, and so was not included in the final model. Therefore, the selected model had no time-dependent regression coefficients.

Age at diagnosis was included in the model as a non-transformed continuous variable. Accurate modeling of the key predictor, thickness, was critical and was done in two stages. First, a smooth rank transform (SRT_thickness) was generated using the *acd* command in Stata [27] and further transformed for inclusion in the model (SRT_thickness^2^). Lack of fit was still apparent, so the original continuous thickness term was also included. The resulting analysis of martingale residuals showed satisfactory fit (results not shown).

### Concordance and discrimination

The effect of each variable on the discrimination (D) and explained variation (${R}_D^2$) are shown in Table 2. The final model explained 49% of the variation, with a D statistic of 1.55. Clearly, the majority of this performance was provided by the transformed thickness variables, with thickness alone accounting for nearly 41% of the variance and a D statistic of 1.32. However, when the remaining variables were combined, excluding thickness, they were able to explain similar amounts of the survival (${R}_D^2 = 0.44,\ \mathrm{D} = 1.40$) as thickness alone. This result suggests relatively high correlation among the predictors. As variables entered as single variables in a model, the most discriminative variables were (in order) thickness, Clark’s level and metastasis. However, when removed from the full model (Table 2), Clark’s level (D: 0.005 and ${R}_D^2:\ 0.002$), morphology (D: 0.008 and ${R}_D^2:\ 0.003$) and gender (D: 0.013 and ${R}_D^2:\ 0.006$) decreased the discrimination by small amounts and the explained variation by less than one percent. The BIC statistic was minimized by excluding Clark’s level and Morphology. Finally, Clark’s level, morphology and gender were removed to form the “parsimonious” model, hereby referred to as the Melanoma Severity Index (MSI).

**Table 2:** **Effect of removing and adding variables from the prognostic model (Probit model with 3df) on discrimination (D) and explained variation (** ${\boldsymbol{R}}_{\boldsymbol{D}}^{\mathbf{2}}$ **)**

	Removing variable					Adding variable				
Variable	Single^a^		Cumulative^b^			Single variable only			Added to thickness	
	D	${\boldsymbol{R}}_{\boldsymbol{D}}^{\mathbf{2}}$	Order	D	${\boldsymbol{R}}_{\boldsymbol{D}}^{\mathbf{2}}$	D	${\boldsymbol{R}}_{\boldsymbol{D}}^{\mathbf{2}}$	Harrell’s C	D^d^	${\boldsymbol{R}}_{\boldsymbol{D}}^{\mathbf{2}}$ ^d^
<full model>	1.526	0.478								
Clark’s level	1.521	0.476	1	1.521	0.476	1.108	0.325	0.815	0.006	0.002
Morphology	1.519	0.475	2	1.513	0.473	0.643	0.140	0.699	0.017	0.006
Gender	1.513	0.473	3	1.500	0.469	0.277	0.029	0.569	0.038	0.014
Age^c^	1.504	0.470	4	1.467	0.458	0.398	0.058	0.646	0.042	0.015
Positive lymph nodes	1.504	0.470	5	1.457	0.451	0.990	0.278	0.541	0.038	0.014
Ulceration	1.503	0.470	6	1.409	0.438	0.998	0.281	0.715	0.044	0.016
metastasis	1.494	0.467	7	1.358	0.420	1.068	0.309	0.530	0.053	0.019
Body site	1.490	0.466	8	1.307	0.401	0.303	0.035	0.600	0.052	0.019
Thickness^c^	1.354	0.419	9	-	-	1.307	0.401	0.866		

^a^Based on the prognostic regression model after removing only the given variable.

^b^Based on the prognostic regression model after sequentially removing the variables in the stated order (1 = first, 2 = second, ..).

^c^Treated as transformed continuous variables (see text for details). The remaining variables are categorical.

^d^Values represent the difference in fit statistics between the model with thickness only ^c^and the model including thickness ^c^and the shown variable.

The Harrell-C statistic for the full model was 0.89 (95% CI = 0.890, 0.894), and 0.88 (0.877, 0.892) for the MSI. Generally the Harrell-C statistics for the individual variables were strongly correlated with the D and ${R}_D^2$ statistics (Table 2), with the exception of metastases and positive lymph nodes. Both these variables had low prevalence (Table 1). Additional unpublished sensitivity analyses suggested that the performance of the Harrell-C statistic in quantifying predictive ability of a variable depends on the prevalence of the feature in question.

### Final parameter estimates

The parameter estimates from the full and the MSI multivariable probit regression model are shown in Table 3. The interpretation of the coefficients from this model is less familiar in a medical context than, for example, the coefficients from linear or logistic regression.

**Table 3:** **Multivariable parameter estimates from the Full and MSI models** ^a^

Variable	Beta coefficient [95% confidence interval]	
	Full model	MSI model
Gender		
Male	*0.000* ^a^	Not included
Female	−0.147 [−0.207, −0.086]	
Age at diagnosis (transformed)	0.007 [0.005, 0.008]	0.007 [0.005, 0.009]
Thickness (original)	0.044 [0.030, 0.058]	0.038 [0.025, 0.050]
SRT_Thickness (transformed)^b^	1.593 [1.394, 1.792]	1.830 [1.697, 1.963]
Body site		
Scalp	0.376 [0.233, 0.519]	0.348 [0.210, 0.486]
Face/Lip/Eyelid	−0.063 [−0.173, 0.046]	−0.110 [−0.215, −0.004]
Ear/Neck	−0.025 [−0.141, 0.091]	−0.041 [−0.156, 0.073]
Chest/Axilla	−0.108 [−0.250, 0.033]	−0.102 [−0.244, 0.039]
Abdomen + Hip	0.148 [−0.010, 0.307]	0.143 [−0.015, 0.301]
Back/Buttocks	*0.000* ^a^	*0.000* ^a^
Upper arm/Forearm/Hand/Finger/sb.hand^c^	−0.321 [−0.425, −0.218]	−0.361 [−0.463, −0.259]
Foot/sb.foot/heel/toe^c^	0.174 [−0.031, 0.378]	0.089 [−0.089, 0.267]
Thigh/leg/ankle	−0.251 [−0.339, −0.163]	−0.291 [−0.376, −0.206]
Shoulder	−0.079 [−0.187, 0.029]	−0.099 [−0.207, 0.009]
Morphology		Not included
SSM	*0.000* ^a^	
Nodular melanoma	0.076 [−0.005, 0.158]	
MM in junctional naevus	0.085 [−0.154, 0.325]	
Lentigo Maligna melanoma	−0.084 [−0.244, 0.077]	
Acral lentiginous melanoma	−0.081 [−0.402, 0.241]	
Other specified melanoma	−0.232 [−0.355, −0.109]	
Malignant melanoma NOS^d^	0.014 [−0.057, 0.086]	
Ulceration		
No ulceration	*0.000* ^a^	*0.000* ^a^
Known ulceration	0.357 [0.277, 0.437]	0.378 [0.300, 0.456]
Positive lymph nodes		
None	*0.000* ^a^	*0.000* ^a^
At least one	0.753 [0.593, 0.912]	0.779 [0.620, 0.938]
Metastasis		
No	*0.000* ^a^	*0.000* ^a^
Yes	1.062 [0.861, 1.262]	1.062 [0.861, 1.262]
Clarks Level		
Into papillary dermis	*0.000* ^a^	Not included
Filling papillary dermis	0.153 [0.052, 0.253]	
Into reticular dermis	0.193 [0.072, 0.314]	
Into subcutaneous fat	0.197 [0.024, 0.370]	

Note: ^a^denotes the reference category.

^b^SRT_Thickness is the smooth rank transform of the thickness variable (see Methods for details).

^c^sb = subungal; ^d^NOS = Not otherwise specified.

A one-unit change in a covariate results in a one-beta change in risk on the probit (inverse normal probability) scale, where beta is the regression coefficient for the variable in question. However, in a more general sense, a positive beta coefficient means that an increase in the covariate raises the predicted probability of death from melanoma. Conversely, a negative beta coefficient means that an increase in the covariate reduces the predicted probability of death.

The full model shows that the probability of death from melanoma was higher among males, older patients, those with thicker melanomas, lesions diagnosed on the scalp, abdomen/hip and foot, nodular melanomas, those with known ulceration, those with positive lymph nodes, evidence of distance metastasis and those with increasing level of invasion (Table 3). Similar direction and magnitude of effects for the included variables were observed for the MSI model (Table 3).

### Internal-external validation

**Figure 3:**
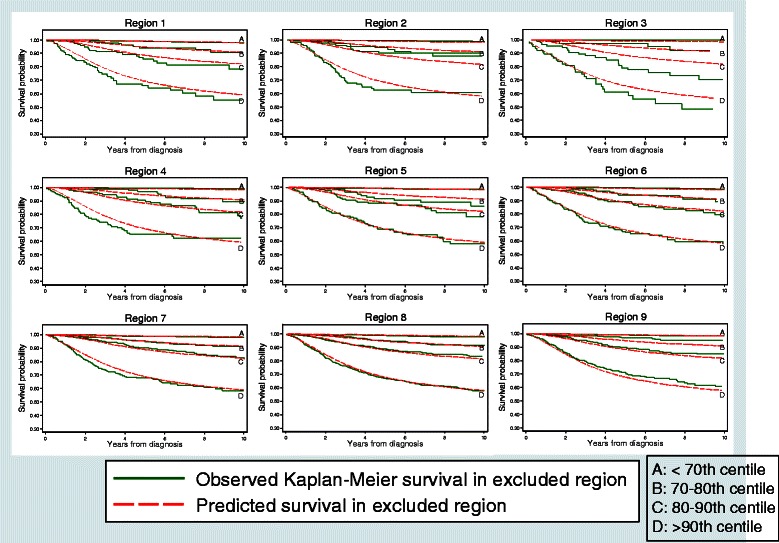
**Predicted and observed survival curves by region from the internal–external cross-validation approach.**

The results of our internal-external cross validation (Figure 3, Table 4) suggests there is limited heterogeneity of the discrimination of the MSI model across the nine geographical regions of Queensland. The only exception was for Region 3, for which the model based on the rest of Queensland had higher discrimination when applied to this region. The mean survival curves predicted by the model generally agreed well with the observed (Kaplan-Meier) survival curves (Figure 3).

**Table 4:** **Evaluation of heterogeneity of the measure of separation (Discrimination, D) across geographically defined regions: internal–external cross-validation using the MSI model**

Region (k)^1^	N_k_	Melanoma deaths_k_	Other deaths_k_	D_(k)_Omitting region k	D_k_Predicted in region k	d_k_(= D_k_– D_(k)_)	SE (d_k_)
1	1,294	84	150	1.50	1.57	0.07	0.14
2	1,438	80	151	1.50	1.51	0.01	0.14
3	919	54	101	1.49	2.34	0.85	0.24
4	1,574	91	192	1.49	1.73	0.24	0.14
5	1,461	104	204	1.50	1.52	0.01	0.13
6	2,313	138	287	1.49	1.75	0.26	0.12
7	3.495	216	446	1.53	1.38	−0.15	0.08
8	10,578	601	1,376	1.52	1.46	−0.06	0.06
9	3,982	212	489	1.53	1.40	−0.13	0.09

^1^Cases for which the geographical region was undefined (n = 1,600, 120 melanoma deaths, 373 other deaths) were retained in the validation process.

### Examples of model predictions for individual patients

**Figure 4:**
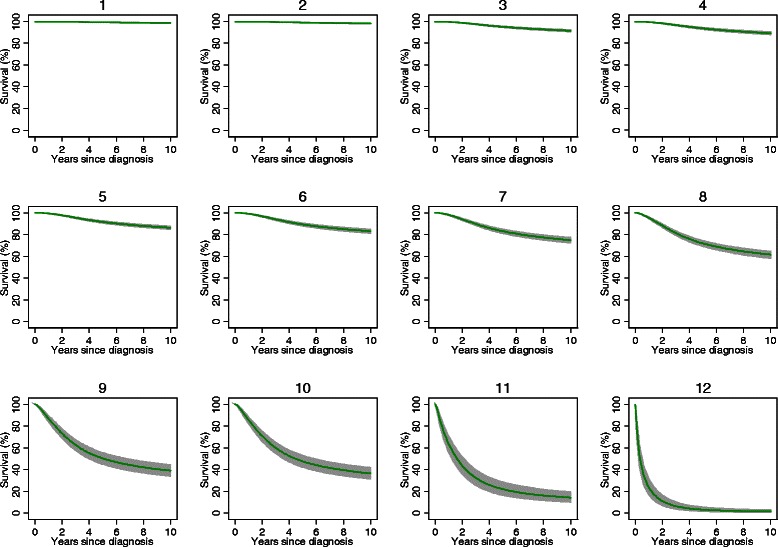
**Predicted 10-year mean survival curves, for twelve hypothetical melanoma patients.**

Examples of the estimated survival probabilities generated by the MSI for twelve hypothetical patients presenting with specific combinations of clinical characteristics and demographics are shown in Table 5 (and Figure 4), with comparison estimates generated by the AJCC Melanoma Patient Outcome Prediction Tool [32]. For example, the estimated 10-year survival of a 55 year old person diagnosed with a 0.45 mm thick melanoma on the back with no evidence of ulceration, positive lymph nodes or metastasis (Example 2) was 98.3%. In comparison, a the 10 year survival for a 75-year old person diagnosed with a 2.45 mm thick melanoma on the back with evidence of ulceration, but no positive lymph nodes or metastasis (Example 8) was 61.4%.

**Table 5:** **Predicted 10-year survival percentages for twelve hypothetical melanoma patients using the MSI, including comparisons with the AJCC Melanoma Patient Outcome Prediction Tool (MPOPT)** ^a^

Characteristic							Estimated survival (%) after ten years	
	Age	Thick (mm)	Site	Ulceration	Positive lymph nodes	Mets	MSI	MPOPT^a^
1.	35	0.45	Back	No	No	No	98.8 [99–99]	97.5 [97–99]
2.	55	0.45	Back	No	No	No	98.3 [98–99]	97.5 [97–99]
3.	55	1.00	Back	No	No	No	91.7 [91–93]	90.0 [88–92]
4.	75	1.00	Back	No	No	No	89.4 [88–91]	82.8 [78–88]
5.	55	1.45	Back	No	No	No	86.2 [85–88]	83.7 [82–86]
6.	75	1.45	Back	No	No	No	83.0 [81–85]	69.6 [63–77]
7.	75	2.45	Back	No	No	No	74.8 [72–77]	59.6 [51–69]
8.	75	2.45	Back	Yes	No	No	61.4 [58–64]	42.0 [33–54]
9.	75	4.45	Scalp	Yes	No	No	38.9 [34–44]	35.2 [27–47]
10.	85	4.45	Scalp	Yes	No	No	36.3 [31-42]	35.2 [27–47]
11.	75	4.45	Scalp	Yes	Yes	No	14.5 [10–20]	29.1 [22–39]
12.	75	4.45	Scalp	Yes	Yes	Yes	1.7 [1–3]	15.9 [6–44]

Notes: ^a^For comparison with the MPOPT (http://www.melanomaprognosis.org), Scalp and Back were categories as “Axial”, and positive lymph nodes or metastasis were both considered indicative of “Regional metastasis).

When comparing the MSI and the MPOPT (Table 5), the predicted 10-year survival percentages for MSI were generally higher than for the MPOPT, with the exception of the two most advanced cases of melanoma.

## Discussion

This prognostic model for people diagnosed with invasive melanoma was developed using a large population-based dataset containing information about the recognized prognostic features of melanoma with up to 16 years of follow-up. The flexible parametric survival model used for this development has many advantages over the standard Cox model used by previous studies, and the population-based registry cohort removed the inherent biases associated with cohorts based on hospital clinic patients. In addition, by extracting additional clinical features from pathology forms, we were able to include clinical factors such as ulceration, number of positive lymph nodes and the presence of metastasis that are not typically available in these population-based data.

There are several benefits of a prognostic model over and above those provided by standard Kaplan-Meier estimates. First, by incorporating demographic and clinical factors, the MSI output can be specifically targeted to an individual. Comparable Kaplan-Meier statistics would need to be generated from stratified subsamples, typically containing insufficient numbers to generate reliable estimates. Second, by incorporating the flexible parametric approach to the prognostic model, it provides the ability to generate other estimates of survival, including longer-term survival, conditional survival and the predicted life expectancy following a melanoma diagnosis.

The single most important feature in the prognostic model was thickness. This has been shown to be an important predictor in previous models, [11,13,32,33] even when restricted to localized [13] or thin [2] melanomas. The MSI can be used to highlight the importance of detecting melanoma early from a public health perspective by quantifying the expected reduction in survival as the thickness at diagnosis increases.

While this prognostic model has not yet been validated against an external, independent dataset, we have examined the internal reliability by assessing its consistency across a variety of different geographical areas within the state, areas that are characterized by differing health patterns and life expectancy. These unmeasured factors are not considered by the prognostic model, so the consistency of the model’s discriminatory performance across these regions is encouraging. Validation using an external dataset would entail calculating the prognostic index using the parameter estimates from this study cohort applied to the covariate values of the secondary dataset. Similarly, the baseline survival function is calculated in the external dataset using both the parameter vector from this study cohort, together with the (log) time values in the external dataset and the set of spline knots used in the current cohort [34].

We did not have information on mitotic rate, and thus have not been able to examine the effect this had on melanoma survival. Mitotic rate is recognized as an important prognostic factor in the final version of the 2009 AJCC Melanoma Staging and Classification [11]. That this variable was omitted here is a limitation. In addition there are other factors that have been shown to influence melanoma survival that have not been included in this model [33], such as HIV infection, race and socioeconomic status. We also did not have information about concomitant diseases that may have independently lowered predicted survival. Importantly, we did include variables that were demonstrated by others to be significant predictors of melanoma survival by other studies, notably thickness, age, body site and ulceration [13,32]. The use of multiple imputation for missing data assumed that the data are missing at random (MAR). We were not able to rule out that the data are missing not at random (MNAR), and it remains possible that there is some unmeasured characteristic of the treating clinician or pathology laboratory that impacted on the completeness of the registry data. Finally, since we included only patients with one diagnosed melanoma to ensure a greater link between the melanoma characteristics and survival outcome, the predicted survival outcomes would no longer be relevant if a subsequent melanoma was diagnosed.

Given the variation in cohort selection, clinical characteristics and statistical methods, direct comparisons with the results of our study to those published recently [2,13,32] for melanoma are difficult. In particular, the comparisons with the AJCC Melanoma Patient Outcome Prediction Tool (United States) [13,32] demonstrate there are important differences between the two countries. It is unknown whether this is due to the statistical method or to cohort selection, since the MPOPT is based on patients selected from major cancer centres and clinical trial cooperative groups. Alternatively, differences in diagnostic or management practices may have led to important differences in the survival outcomes expected by people diagnosed with melanoma in the two countries.

From a clinical perspective, this MSI could be readily applied to individual melanoma patients. We plan to incorporate a MSI online dissemination tool into a broader package for primary care physicians to assist in discussions about prognosis in the clinical setting.

One of the many advantages of using the flexible parametric modeling approach is that these models can be readily extended to consider estimates of conditional survival [20] and loss of life expectancy [35] due to their diagnosis in comparison to the general population. We have previously shown that people diagnosed with localized or regional melanoma have negligible excess mortality compared to the general population once they have survived ten years post-diagnosis [36,37]. Such models enable more precise estimates of conditional survival by incorporating clinically relevant factors in the estimation. Further work is also planned on the impact of competing risks within the flexible parametric framework [20,38].

We did not find any time-dependent regression coefficients of sufficient impact to include them in the final model. Proponents of other prognostic models using large epidemiological cancer datasets have observed time-varying coefficients [38-40] on a hazard scale. However, in our probit model, hazard ratios comparing any two values of a covariate can be shown to tend toward 1 as follow-up time increases. Thus, all hazard ratios are time-dependent. When there are multiple time-dependent coefficients, interpreting the time dependent hazard ratios can be difficult in the log cumulative hazard framework of the Royston-Parmar models [20]. The reason is that hazard ratios depend on the values of more than just one covariate. Alternatives, including modelling on the log excess hazard scale, may offer more interpretable options when time dependent coefficients are present [41].

## Conclusions

The MSI serves to identify and weight key parameters of prognostic importance in melanoma, and therefore allow a finer gradation of the prognostic value of interventions than the simple dichotomous variable of death due to melanoma, with greater statistical power for comparisons. It also enables predictions of survival outcomes soon after the intervention, rather having a 5 or ten year delay for follow-up. Thus, beyond the immediate clinical use, the MSI may have important public health and research applications for evaluations of public health interventions aimed at reducing deaths from melanoma.

## Abbreviation

MSI: Melanoma severity index
